# Polymethoxyflavones: Novel β-Secretase (BACE1) Inhibitors from Citrus Peels

**DOI:** 10.3390/nu9090973

**Published:** 2017-09-04

**Authors:** Kumju Youn, Yoonjin Yu, Jinhyuk Lee, Woo-Sik Jeong, Chi-Tang Ho, Mira Jun

**Affiliations:** 1 Department of Food Science and Nutrition, Dong-A University, 37, Nakdong-daero 550 beon-gil, Saha-gu, Busan 49315, Korea; kjyoun@dau.ac.kr (K.Y.); yoonjiinyou@daum.net (Y.Y.); 2 Korean Bioinformation Center, Korea Research Institute of Bioscience and Biotechnology, 125, Gwahak-ro, Yuseong-gu, Daejeon 34141, Korea; mack97hyuk@gmail.com; 3 Department of Bioinformatics, University of Sciences and Technology, 100, Hyecheon-ro, Seo-gu, Daejeon 34141, Korea; 4 Department of Food & Life Science, College of Biomedical Science & Engineering, Inje University, 197, Inje-ro, Gimhae-si, Gyeongsangnam-do 50834, Korea; jeongws@inje.ac.kr; 5 Department of Food Science, Rutgers University, New Brunswick, NJ 08901, USA; ho@aesop.rutgers.edu

**Keywords:** Alzheimer’s disease (AD), β-secretase (BACE1), citrus peel, polymethoxyflavones (PMFs)

## Abstract

Beta-site amyloid precursor protein (APP) cleaving enzyme1 (BACE1) catalyzes the rate-limiting step of amyloid-β protein (Aβ) generation, and is considered as a prime target for Alzheimer’s disease (AD). In search of a candidate for AD prevention, our efforts exploring the natural BACE1 inhibitor have led to the finding of nobiletin, tangeretin, and sinensetin—representative compounds of polymethoxyflavones (PMFs). Tangeretin exhibited the strongest BACE1 inhibition (IC_50_, 4.9 × 10^−5^ M), followed by nobiletin and sinensetin with IC_50_ values of 5.9 × 10^−5^ M and 6.3 × 10^−5^ M, respectively. In addition, all compounds reacted in a non-competitive manner with the substrate. Docking analysis results for complexes with BACE1 indicated that SER10 and THR232 residues of BACE1 hydrogen bonded with two oxygen atoms of tangeretin, while three additional BACE1 residues (ALA157, VAL336 and THR232) interacted with three oxygen atoms of nobiletin. Furthermore, sinensetin formed four hydrogen bonds through nitrogen atoms of TYR71, LYS75, and TRP76, and an oxygen atom of TYR198. Furthermore, the lowest-energy conformations of the most proposed complexes of sinensetin, nobiletin, and tangeretin with BACE1 were −7.2, −7.0, and −6.8 kcal/mol, respectively. Taken together, our results suggest that these polymethoxyflavones (PMFs) might be considered as promising BACE1 inhibitory agents that could lower Aβ production in AD.

## 1. Introduction

Alzheimer’s disease (AD) is a devastating neurodegenerative disorder that alters the mental capacity of patients suffering from the disease [1]. Pathologically, AD occurs mainly because of the generation of two hallmark lesions—neurofibrillary tangles and amyloid plaques—in the brain. Neurofibrillary tangles are insoluble bundles of fibers which are usually made up of phosphorylated tau protein [2]. Amyloid plaques are spherical lesions that contain extracellular aggregates of amyloid-β protein (Aβ). The role of Aβ peptides in the pathogenesis of AD remains unclear; however, several pieces of evidence suggest that an abnormal accumulation of Aβ peptides in the brain is the main cause of AD. Aβ is generated from the amyloid precursor protein (APP) through a two-step proteolytic cleavage. β-Secretase (BACE1) facilitates the first proteolytic step, which releases an N-terminus (sAPPβ) into the extracellular medium. Following β-secretase cleavage, the remaining C99 undergoes further proteolytic cleavage by γ-secretase to generate the C-terminus of Aβ, and the mature peptide is secreted from the cell [3,4]. Therefore, a strategy to delay or prevent AD could be to stop the proliferation of plaques by inhibiting these secretases.

γ-Secretase is a membrane protein complex composed of presenilin 1 or 2 (PS1 or PS2) as the catalytic subunit, Aph-1a or -b, nicastrin (NCT), and presenilin enhancer-2 (PEN-2). PS1 and PS2 are two integral membrane proteins found in the endoplasmic reticulum and Golgi apparatus, and are the key targets for γ-secretase inhibition in the treatment of AD [5]. However, apart from their essential role in generating the Aβ peptide, PS1 and PS2 regulate the Notch signaling pathway responsible for embryonic development. In addition, only a few types of PS1/PS2-null mouse models survive after birth, because PS1/PS2 knockout mice have substantial neuronal deficits, skeletal defects, underdeveloped subventricular areas, and severe hemorrhages [6,7,8]. 

BACE1 knockout mice were initially reported to be free of negative phenotypes; subsequent investigations identified BACE1 null abnormalities such as axon guidance defects, hypomyelination, memory deficits, spin density reduction, impairment of synaptic plasticity, neurogenesis and astrogenesis abnormalities, etc., suggesting that BACE1 inhibitors might produce mechanism-based side-effects [9]. However, the risk of BACE1 mechanism-based toxic effects might depend on the level of BACE1 inhibition. A previous study of BACE1 heterozygote mice overexpressing mutant human APP platelet-derived growth factor promoter (PDAPP;BACE1+/−) showed a considerable reduction in brain Aβ levels and plaque load without side effect [10]. A further study used heterozygous BACE1 gene knockout (BACE1+/−) mice to demonstrate that a 50% BACE1 reduction is sufficient to rescue deficits in brain function without any abnormal effect in an AD transgenic mouse model [11]. Experimental observation from mouse models of AD indicate that a level of BACE1 inhibition between 50% and 75% could be sufficient to reduce the rate of Aβ and prevent amyloid deposition without side effect. Clinical programs today target intermediate steady-state inhibition levels such as 50% or 85% BACE1 inhibition [12]. Therefore, potential mechanism-based side effects might occur only in completely abolishing BACE1 activity, but not in partially inhibiting it. Based on these findings, despite these cautionary notes, the inhibition of BACE1 activity could be a promising molecular target for lowering Aβ in AD.

Recently, much attention has been paid to the screening of products from natural sources, because they are usually considered to be less toxic and have fewer side effects than products from synthetic sources. In particular, the wide biochemical functions of polymethoxyflavones (PMFs) have been studied extensively. They are of particular interest because of their broad range of biological activities, including antioxidant, anticarcinogenic, and anti-inflammatory properties [13,14,15]. PMFs are found almost exclusively in plants of the genus *Citrus*, and are particularly more abundant in the peel than in other edible parts of the fruit [16]. The neuroprotective properties of citrus peel extract (CPE) have been demonstrated in several studies. CPE induced mild mitochondrial depolarization by inhibiting mitochondrial calcium overload in H_2_O_2_-stimulated HT-22 neurons [17]. In addition, CPE facilitated cyclic adenosine monophosphate/protein kinase A/extracellular signal-regulated kinase/cAMP response element binding (cAMP/PKA/ERK/CREB) signaling associated with learning and memory in cultured hippocampal neurons [18]. Nobiletin (5,6,7,8,3′,4′-hexamethoxyflavone), tangeretin (5,6,7,8,4′-pentamethoxyflavo-ne), and sinensetin (5,6,7,3′,4′-pentamethoxyflavone) are the most common PMFs found in citrus peel extract. Although the beneficial effects of the PMFs have been reported previously, the potential of their inhibitory activities against BACE1 in preventing and/or treating AD was first evaluated in this study. In the present study, the activities of nobiletin, tangeretin, and sinensetin as BACE1 inhibitors were assessed, and in silico docking analysis was performed to determine their specific binding sites and lowest binding energies with respect to human BACE1.

## 2. Materials and Methods 

### 2.1. General

Nobiletin (>98% purity) was purchased from Santa Cruz Biotechnology (Santa Cruz, CA, USA). Tangeretin, sinensetin, (>95% purity), and resveratrol (>99% purity) were obtained from Sigma-Aldrich (St. Louis, MO, USA). BACE1 inhibition was determined by enzymatic assay using the fluorescent resonance energy transfer (FRET)-based BACE1 kit from Invitrogen (Pan Vera, Madison, WI, USA). α-Secretase (tumor necrosis factor-α converting enzyme, TACE) and substrate were obtained from R&D Systems (Minneapolis, MN, USA). Trypsin, chymotrypsin, elastase, and their substrates were obtained from Sigma-Aldrich (St. Louis, MO, USA). The measurement of fluorescence and optical density was performed with a BioTEK ELISA microplate fluorescence reader FLx 800 and BioTEK ELx 808 (Winooski, VT, USA), respectively.

### 2.2. Enzymatic Assessment for Biological Evaluation

BACE1, TACE, chymotrypsin, trypsin and elastase assays were performed according to previous methods [19]. Fluorometric BACE1 and TACE assay were measured using a Rh-EVNLDAEFK-Quencher and Mca-PLAQAV-Dpa-RSSSR-NH_2_ as substrates. Trypsin, chymotrypsin, and elastase were assayed according to the manual described in the reference using *N*-benzoyl-l-Arg-pNA, *N*-benzoyl-l-Tyr-pNA, and *N*-succinyl-Ala-Ala-Ala-pNA as substrates, respectively.

### 2.3. Assessment of the Inhibition Kinetics on BACE1

The kinetic mechanisms of the different samples towards BACE1 were determined by the graphical views of Dixon and Lineweaver–Burk plots. The inhibitory constants (Ki) value was defined by interpretation of the Dixon plot, where the value of the x-axis implies -Ki. Maximum velocity (Vmax) and Michaelis constant (Km) were obtained by Lineweaver–Burk plots, using initial velocities obtained over a substrate concentration ranging from 250 to 750 nM. The kinetic parameters were then calculated using Enzyme Kinetic^TM^ module of SigmaPlot^TM^ version 12.3 (Systat Software, Inc., San Jose, CA, USA).

### 2.4. In Silico Docking Studies

A computational ligand–target docking method was used to investigate structural complexes of the BACE1 (target) with PMFs (ligand) in order to understand the structural basis of this protein target specificity [20]. Specifically, we used Autodock Vina to dock different compounds into the binding pocket residue of the BACE1 crystallographic structure, which was defined as all residues 5 Å from the inhibitor in the original complex. For docking analysis, the crystal structure of the BACE1 protein target was prepared from the protein sequence alignment (Protein Data Bank (PDB ID 2WJO)) and for nobiletin, tangeretin and sinensetin were obtained from the PubChem database (CID 72344, 68077 and 145659, respectively). Chemical structures were drawn and displayed using Marvin (5.11.4, 2012, ChemAxon, One Broadway, Cambridge, MA, USA) [21]. All docking structures were clustered and categorized by the lowest energy and the largest number of clusters.

### 2.5. Statistical Analysis

All experiments were presented as the mean ± standard deviation (SD) of three independent experiments. Significant differences were conducted by Duncan’s multiple range tests using Statistical Analysis System (SAS) version 9.3 (Cary, NC, USA).

## 3. Results

### 3.1. In Vitro BACE1 Inhibitory Activity of Biochanin A

**Figure 1:**
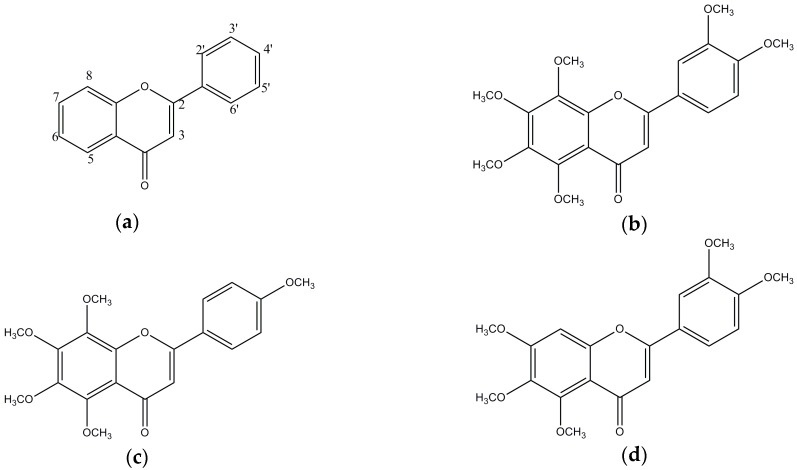
The chemical structures of polymethoxyflavones (PMFs): (**a**) flavone; (**b**) nobiletin; (**c**) tangeretin; (**d**) sinensetin.

**Figure 2:**
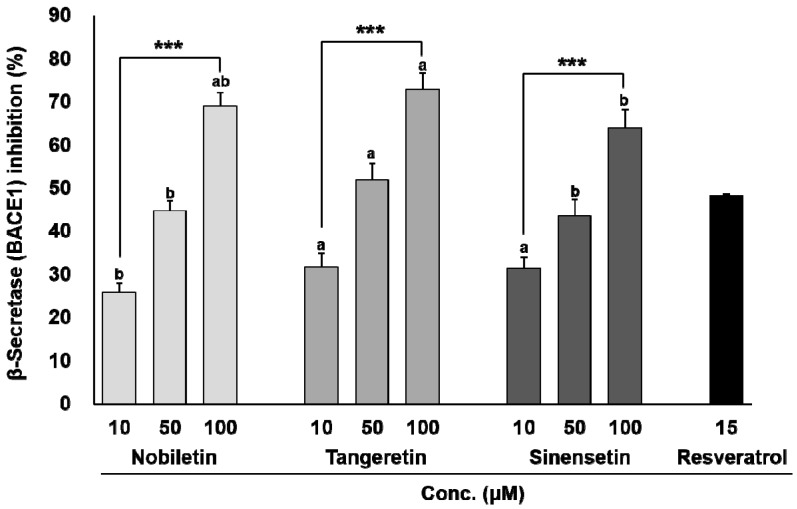
β-Secretase (BACE1) inhibitory activities of polymethoxyflavones (PMFs). The activities (%) are expressed as mean ± standard deviation (SD) of three independent experiments. Each concentration of the same compounds is significantly different at *** *p* < 0.001. The same concentrations of each compound with different letters are significantly different at *p* < 0.001.

The chemical structures of nobiletin, tangeretin, and sinensetin are shown in Figure 1. As shown in Figure 2, the three compounds blocked BACE1 in a dose-dependent manner (*p* < 0.001). Tangeretin had the highest BACE1 inhibitory property (IC_50_, 4.9 × 10^−5^ M), followed by nobiletin (IC_50_, 5.9 × 10^−5^ M) and sinensetin (IC_50_, 6.3 × 10^−5^ M). The common structures of nobiletin, tangeretin, and sinensetin include three methoxy groups at C5, C6, and C7 in the A ring and one methoxy group at C4′ in the B ring, which provide a partial BACE1-suppressive potency. Interestingly, the presence of C3′-OCH_3_ in the B ring in nobiletin and sinensetin reduced their inhibitory potency. However, an additional C8-OCH_3_ in the A ring of tangeretin noticeably enhanced its anti-BACE1 activity. Therefore, the C8-OCH_3_ in the A ring was considered an enhancer of the anti-BACE1 activity, whereas the anti-BACE1 activity decreased in the presence of C3′-OCH_3_ in the B ring.

To prove the enzyme specificity of PMFs against BACE1, their inhibitory activities against BACE1 were compared with their inhibitory activities against TACE and other serine proteases (e.g., trypsin, chymotrypsin, and elastase) (Table 1). None of the tested compounds showed statistically significant inhibition against TACE or other serine proteases, suggesting that nobiletin, tangeretin, and sinensetin are specific inhibitors of BACE1.

### 3.2. BACE1 Kinetic Assay

**Figure 3:**
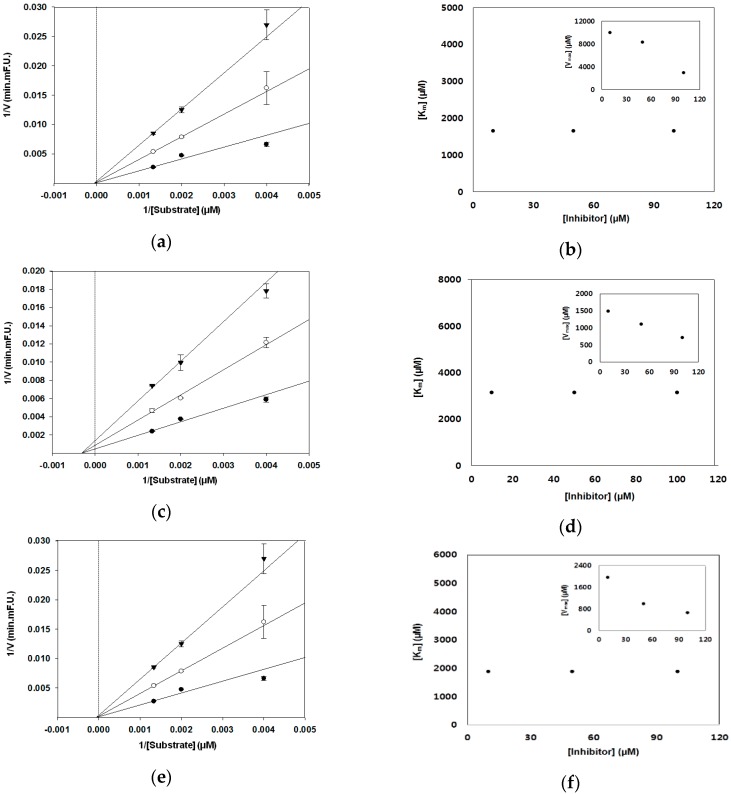
Lineweaver–Burk plot of the reciprocal initial velocities of (**a**) nobiletin, (**c**) tangeretin, and (**e**) sinensetin against BACE1 concentration at different polymethoxyflavone (PMF) concentrations: 10 M (●); 50 μM (○); 100 μM (▼). The Km values as a function of the concentration of (**b**) nobiletin, (**d**) tangeretin, and (**f**) sinensetin. Insets in (**b**,**d**,**f**) show the dependence of the values of Vmax on the concentration of PMFs.

**Figure 4:**
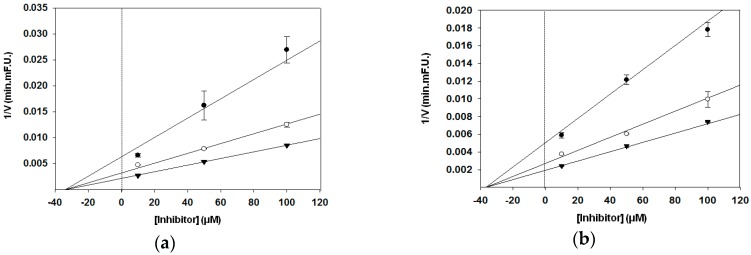

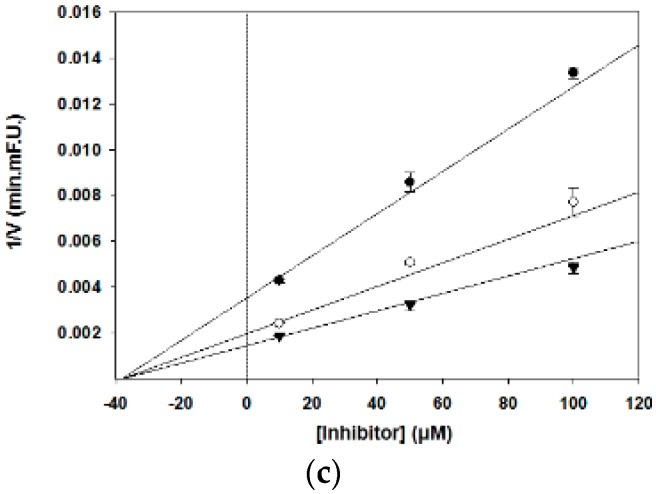
Dixon plot of the reciprocal initial velocities of (**a**) nobiletin, (**b**) tangeretin, and (**c**) sinensetin against BACE1 concentration at several fixed substrate concentrations: 250 nM (●); 500 nM (○); 750 nM (▼).

In the present study, different graphical analyses were carried out to distinguish the type of inhibition by PMFs. Analysis of kinetic parameters obtained from the Dixon and Lineweaver–Burk plots showed that nobiletin, tangeretin, and sinensetin are noncompetitive inhibitors. These compounds decreased the Vmax of FRET substrate decomposition reaction by BACE1, whereas the Km value remained unchanged in the Lineweaver–Burk plot (Figure 3). As shown by the Dixon plot, a change in the slope and y-intercept of the curve was observed in the presence of an inhibitor, but the x-intercept remained unchanged (Figure 4). The obtained Ki values calculated from the Dixon plots were 3.4 × 10^−5^ M for nobiletin, 3.7 × 10^−5^ M for tangeretin, and 3.8 × 10^−5^ M for sinensetin. A lower Ki value represents a stronger bond between an inhibitor and an enzyme, thus implying greater effectiveness of the inhibitor. Thus, our present results suggest that PMFs could be good BACE1 inhibitors.

### 3.3. Molecular Docking Study of the Inhibitory Activity of PMFs against BACE1

**Figure 5:**
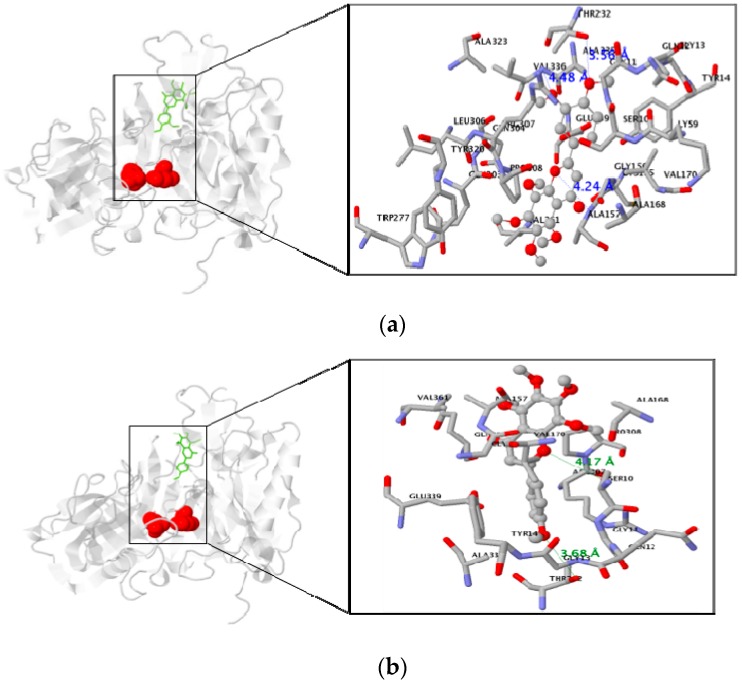

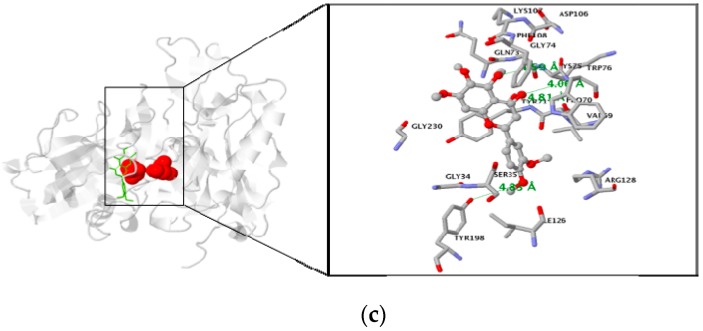
Comparison of in silico docking poses for polymethoxyflavones (PMFs): (**a**) nobiletin, (**b**) tangeretin, (**c**) sinensetin. Representative binding mode of the most stable docking poses of PMFs with BACE1. BACE1 is expressed as a solid ribbon diagram and PMFs as a stick depiction. Left: The complete view of the docking pose of PMFs. Right: The close-up view of the calculated PMFs–BACE1 docking modes. Asp32 and Asp228 are the active catalytic center residues shown in italics. Hydrogen bond interactions between PMFs and the human BACE1 residues are presented as blue (**a**) and green dots (**b**,**c**).

The AutoDock program uses a semi-empirical energy force field to guess the binding of protein–ligand complexes of known structure and binding energies. The docking results of the BACE1–PMFs complexes revealed that nobiletin, tangeretin, and sinensetin were stably positioned in allosteric sites of the BACE1 residues, all of which were 5 Å from the inhibitor in the original complex (Figure 5). The oxygen atoms of nobiletin formed three hydrogen bonds with two nitrogen atoms of ALA157 and VAL336 and one oxygen atom of THR232 in BACE1 (distance: 4.24, 4.48, and 3.56 Å, respectively). In comparison, tangeretin had only two hydrogen bonds with oxygen atoms of SER10 and THR232 (distance: 4.17 and 3.68 Å, respectively). Sinensetin formed four hydrogen bonds with nitrogen atoms of TYR71, LYS75, and TRP76, and with an oxygen atom of TYR198 (distance: 4.81, 4.59, 4.06, and 4.86 Å, respectively). In addition, the lowest binding energies of PMFs were negative values: −7 kcal/mol for nobiletin, −6.8 kcal/mol for tangeretin, and −7.2 kcal/mol for sinensetin (Table 2).

## 4. Discussion

Recently, PMFs were shown to possess neuroprotective effects in both cell and animal models. Nakajima et al. and Nagase et al. reported that the administration of nobiletin for 11 days significantly recovered olfactory bulbectomy-induced memory impairment [22,23]. In addition, APP-SL 7-5 Tg mice were administered nobiletin daily from 4 to 9 months of age, and it significantly reversed memory impairment without affecting general behavior in the context-dependent fear conditioning test and reduced quantity of Aβ_1-42_ and Aβ_1-40_ in the brain [24]. In addition, nobiletin reduced the levels of both soluble Aβ_1-40_ and reactive oxygen species (ROS) in the brain and in the hippocampus of 3XTg-AD mice [25]. Tangeretin reportedly suppressed LPS-induced primary rat microglia and BV-2 microglial cell activation by modulating the mitogen-activated protein kinase and NF-κB signaling pathways [26]. Subchronic treatment of rats with tangeretin (20 mg/kg/day) for 4 days before 6-oxidopamine (OHDA) injection markedly reduced the loss of both TH+ cells and striatal dopamine content induced by unilateral infusion of 6-OHDA to the medial forebrain bundle [27]. Sinensetin has been studied to a lesser extent than nobiletin and tangeretin, but it was recently shown to activate cyclic AMP response element-mediated transcription in rat hippocampal neurons [18]. 

Although not fully understood, the metabolites of PMFs have been studied, and it was found that they undergo in vivo biotransformation, producing metabolites with different bioactivities and pharmacological properties. The bioinformation study of nobiletin has shown that the compound undergoes a demethylation pathway with the formation of mono-demethyl nobiletin (DMN) as major metabolites such as 3′-, 4′-, 6-, or 7-DMN. Through di-demethylation, nobiletin transforms to 3′, 4′-di-DMN or 6, 7-di-DMN [28,29]. Like nobiletin, the major nobiletin metabolite 4′-DMN was revealed to stimulate phosphorylation of ERK and CREB signaling pathways related to memory process, and was further shown to be able to cross the blood–brain barrier (BBB) [30]. In addition, 3′-DMN inhibited inducible nitric oxide synthase (iNOS) more efficiently than nobiletin, whereas other two metabolites 4′-DMN and 3′,4′-di-DMN more efficiently suppressed the gene expression of iNOS and cyclooxygenase-2 (COX-2) than their parent compound [31]. As major metabolites of tangeretin, 4′-demethyltangeretin and 3′,4′-, 7,4′-, or 6,7-demethyltangeretin have been detected [32], and four metabolites of sinensetin (i.e., 4′-, 5-, or 6-OH sinensetin and 7-OH sinensetin sulfate) were identified [33]. Study of the biological actions of the metabolites of both tangeretin and sinensetin are lacking, but it is likely that biological actions similar to nobiletin and its metabolites might also be attributable to these compounds.

Despite the evidence for PMF-mediated neuroprotection, bioavailability in the brain is important for active compounds to be effective in therapeutic for AD, because an inability to cross the blood–brain barrier (BBB) can limit the prevention and/or treatment applications of compounds [34]. When nobiletin was orally administered (50 mg/kg), the content of this compound was revealed to be 3.6 mg/kg (approximately 8.9 µM) in the brains of mice [35]. The previous study also demonstrated nobiletin accumulation in the brain of A/J mice fed nobiletin (250, 500, or 1000 ppm in diet for 16 weeks), which suggested that nobiletin may absorbed and penetrate BBB into the brain tissues where it may directly act as a therapeutic agent on the neural cells. Time-dependent dynamics of nobiletin in the serum and brain showed that nobiletin may rapidly penetrate the BBB and enter the brain tissues, where it may directly act as a therapeutic agent on neural cells, including hippocampal neurons [35]. Interestingly, nobiletin exhibited high accumulation in the brain, which was even much higher than that in the liver [36]. Even though the tested compounds in our study have been revealed to penetrate the BBB and stay active in the brain when orally administered (since they are hydrophobic compounds), orally-consumed PMFs may be poorly absorbed in general. Thus, to overcome the limitation of poor bioavailability of PMFs, several studies have been focused on the formation of amorphous and nanosized nobiletin for enhancing the bioavailability and CNS delivery [37]. Datla and coworkers showed that tangeretin was detected in the hippocampus after chronic oral administration [27]. Furthermore, the multidrug resistance transporter P-glycoprotein (P-gp) is an active component of the BBB, acting as an ATP-driven efflux pump, controlling the movement of structurally diverse molecules across the BBB [38]. Nobiletin and tangeretin have inhibitory potential with P-gp in adriamycin-resistant human myelogenous leukemia cells and in Caco-2 cells using talinolol as a probe [39,40].

Oral acute and chronic toxicity studies are important to determine the safety of drugs and plant products for human use. However, to date, information on the toxicology profile for PMFs is still limited. Ting et al. (2015) [41] reported that rats administered tangeretin up to 1000 to 3000 mg/kg bw showed no evidence of death nor significant change of clinical chemistry in oral acute and sub-acute study. Alterations of the hepatic cell and lipid profile increased dose-dependently and exhibited a distinct injury recovery pattern. The results of a recent pilot clinical study indicated that nobiletin-rich *Citrus reticulata* peel extract treatment for 1 year could prevent the progression of the cognitive impairment in donepezil-preadministered AD patients with no adverse side effects [42]. 

It is important to reiterate that the risk of mechanism-based toxic effects might depend on the level of BACE1 inhibition. Partial inhibition of BACE1 activity could represent a feasible approach. For example, the currently tested BACE1 inhibitor MK-8931 has been safe and tolerated after multiple-dose administration for at least 18 months in human subjects [12]. Since natural BACE1 inhibitors (e.g., PMFs) have relatively weaker BACE1 inhibitory effects than the synthetic one, they may be free from side effects caused by excessive BACE inhibition. Although further pharmacokinetic explanations of PMFs in an animal model are required, this study provides evidence that PMFs exerted significant and specific inhibitory properties against BACE1.

## 5. Conclusions

Our findings suggest that PMFs have a significant inhibitory activity against BACE1, whereas they lack any inhibitory property against TACE and other serine proteases. Enzyme kinetics was evaluated using the Dixon and Lineweaver–Burk plots to identify compound inhibition modes. In addition, molecular docking studies indicated strong hydrogen bonding with several important amino acid residues, as evidenced by negative binding energies at the allosteric site in BACE1; this can explain the potency of these compounds. Although further BACE1 selectivity over cathepsins D and BACE2 and in vivo studies are required to confirm our findings, these PMFs showed significant and selective inhibitory activities against BACE1, and can be used as potential agents for preventing and/or treating AD.

## Figures and Tables

**Table 1:** Inhibitory activities (%) of polymethoxyflavones (PMFs) ^1,2^ against α-secretase (tumor necrosis factor-α converting enzyme, TACE) and other serine proteases

Sample (μM)	TACE	Trypsin	Chymotrypsin	Elastase
Nobiletin				
50	5.49 ± 0.34	1.00 ± 0.07	1.39 ± 0.17	5.51 ± 1.17
100	11.61 ± 3.07	0.92 ± 0.06	1.77 ± 0.06	4.49 ± 1.00
Tangeretin				
50	8.00 ± 1.00	8.10 ± 0.99	5.68 ± 0.86	8.64 ± 0.65
100	11.28 ± 1.66	11.80 ± 1.04	6.16 ± 0.38	10.58 ± 1.09
Sinensetin				
50	7.88 ± 1.12	5.16 ± 0.69	9.08 ± 0.48	7.40 ± 2.09
100	7.19 ± 1.35	4.70 ± 0.58	8.78 ± 0.80	11.41 ± 1.38

^1^ The inhibition (%) of PMFs against TACE, trypsin, chymotrypsin, and elastase is expressed as mean ± SD based on three independent experiments; ^2^ Comparison of concentration level in PMFs is not significantly different.

**Table 2:** Molecular interactions of BACE1 sites with polymethoxyflavones (PMFs).

Ligand	Binding Energy (kcal/mol)	No. of Hydrogen Bonds	Hydrogen Bonds Interacting Residues
Nobiletin	−7.0	3	Residue in 5 Å : ALA157, VAL336, THR232
Tangeretin	−6.8	2	Residue in 5 Å :SER10, THR232
Sinensetin	−7.2	4	Residue in 5 Å : TYR71, LYS75, TRP76, TYR198
